# Predicting volume of distribution with decision tree-based regression methods using predicted tissue:plasma partition coefficients

**DOI:** 10.1186/s13321-015-0054-x

**Published:** 2015-02-26

**Authors:** Alex A Freitas, Kriti Limbu, Taravat Ghafourian

**Affiliations:** School of Computing, University of Kent, Canterbury, CT2 7NF UK; Medway School of Pharmacy, Universities of Kent and Greenwich, Chatham, Kent, ME4 4TB UK; Drug Applied Research Centre and Faculty of Pharmacy, Tabriz University of Medical Sciences, Tabriz, Iran

**Keywords:** Volume of distribution, Tissue partition, QSAR, QSPkR, Data mining, Machine learning, Decision tree, Pharmacokinetics, ADME

## Abstract

**Background:**

Volume of distribution is an important pharmacokinetic property that indicates the extent of a drug’s distribution in the body tissues. This paper addresses the problem of how to estimate the apparent volume of distribution at steady state (Vss) of chemical compounds in the human body using decision tree-based regression methods from the area of data mining (or machine learning). Hence, the pros and cons of several different types of decision tree-based regression methods have been discussed. The regression methods predict Vss using, as predictive features, both the compounds’ molecular descriptors and the compounds’ tissue:plasma partition coefficients (K_t:p_) – often used in physiologically-based pharmacokinetics. Therefore, this work has assessed whether the data mining-based prediction of Vss can be made more accurate by using as input not only the compounds’ molecular descriptors but also (a subset of) their predicted K_t:p_ values.

**Results:**

Comparison of the models that used only molecular descriptors, in particular, the Bagging decision tree (mean fold error of 2.33), with those employing predicted K_t:p_ values in addition to the molecular descriptors, such as the Bagging decision tree using adipose K_t:p_ (mean fold error of 2.29), indicated that the use of predicted K_t:p_ values as descriptors may be beneficial for accurate prediction of Vss using decision trees if prior feature selection is applied.

**Conclusions:**

Decision tree based models presented in this work have an accuracy that is reasonable and similar to the accuracy of reported Vss inter-species extrapolations in the literature. The estimation of Vss for new compounds in drug discovery will benefit from methods that are able to integrate large and varied sources of data and flexible non-linear data mining methods such as decision trees, which can produce interpretable models.

**Graphical Abstract:**
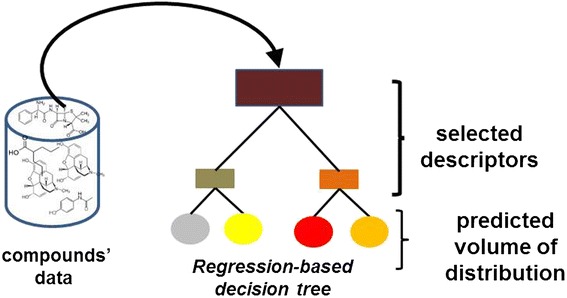
Decision trees for the prediction of tissue partition coefficient and volume of distribution of drugs.

**Electronic supplementary material:**

The online version of this article (doi:10.1186/s13321-015-0054-x) contains supplementary material, which is available to authorized users.

## Background

Despite significant advances in pharmacology in the last decades, at present it is still very difficult to find (near)-optimal answers to the questions of *how much*, *how often* and *for how long* a drug should be given to a patient, in order to maximize its therapeutic effect and minimize its adverse effects. This is especially important in drug discovery since, for a new drug candidate, a poorly designed study with incorrect dose regimen can lead to misleading results which can prove very costly to the sponsor company when the product fails later in development.

In this context, this paper addresses an important pharmacokinetics problem: how to estimate the apparent volume of distribution of a drug in the human body at steady state (Vss), which is the volume of reference fluid (usually plasma) in which the drug appears to be dissolved at steady state [1]. Although this apparent Vss has no physiological meaning, its estimation is important because it predicts the drug’s plasma concentration for a given amount of drug in the body and it influences the drug’s half-life [2,3], which in turn is very important to determine the correct dosage regimen that clinicians should prescribe to patients [4,5]. Hence, one needs to estimate or predict Vss using an *in vivo*, *in vitro* or *in silico* approach [6-9].

*In vivo* animal models produce rich information about a drug’s pharmacokinetics properties, but they are a low-throughput approach which is very time-consuming and costly, as well as involving ethical issues. *In vitro* models are less time-consuming and less costly than *in vivo* ones, but they are still based on time-consuming and costly biological assays, being at best a medium-throughput approach. For a review and comparison of *in vivo* and *in vitro* methods for Vss prediction, see e.g. ref. [10].

*In silico* models are theoretical models that lack the experimentally-derived rich information associated with *in vivo* or *in vitro* models, but they are a very high-throughput approach, which is much less time-consuming and costly than *in vivo* and *in vitro* approaches. Hence, the results of an *in silico* model can be used to suggest which chemical compounds should have a higher priority to be further tested by the more expensive but more accurate *in vivo* and *in vitro* experiments. In addition to much smaller time and cost requirements, *in silico* models have the advantages that they can be directly generated with human data and can even be used to evaluate the pharmacokinetics of compounds which have not been synthesized yet, which is not possible with *in vivo* and *in vitro* experiments.

This work is based on the *in silico* approach, using data mining (or machine learning) methods to build models that predict the Vss using the properties of molecular structures of chemical compounds as the model features. Such a modeling approach is generally known as Quantitative Structure-Activity Relationship (QSAR) approach, with the special QSAR case here being the Quantitative Structure-Pharmacokinetic Relationship (QSPkR) modeling [11,12]. More precisely, we use two types of data mining methods – mainly decision tree-based regression methods, but also a feature selection method (see Methods section) – to produce QSPkR models that predict the Vss of chemical compounds.

Conventional QSPkR modeling methods for predicting Vss normally use, as features, a large set of physicochemical or molecular descriptors, most of which are calculated by specialized software. The use of such physicochemical descriptors as potential predictors of Vss makes sense because, broadly speaking, the Vss of a drug is mainly determined by its nonspecific binding to plasma and tissue components, rather than its specific pharmacophore, and such nonspecific binding is to a large extent determined by the drug’s physicochemical properties [4,13,14]. In addition, binding to the pharmacological target is considered to have relatively little importance for predicting Vss, since the level of target expression is usually low [15].

As an alternative to building QSPkR models for predicting Vss based on physicochemical drug properties, several studies use a physiologically-based pharmacokinetics (PBPK) approach for predicting the Vss of a drug based on its tissue:plasma partition coefficients (K_t:p_), where a compound’s K_t:p_ is its concentration ratio between a tissue and plasma at steady state. The basic idea is to determine the K_t:p_ value for each of the major tissues in the body where a drug can be present in significant concentration and calculate the Vss for the whole body as a function of the sum of the product of K_t:p_ and tissue volume for all those tissues, using (variations of) the following equation [16,17]: *Vss* = *V*_*p*_ + *V*_*e*_ × (*E:P*) + Σ_*t*_ (*V*_*t*_ × *K*_*t:p*_), where V_p_, V_e_ and V_t_ are the volumes of plasma, erythrocyte and tissue, respectively, *E:P* is erythrocyte-to-plasma partition coefficient, and K_t:p_ is the tissue:plasma partition coefficient for tissue t. Note that this equation refers to K_t:p_ coefficients based on total concentrations in tissue and plasma, but one could use instead coefficients referring to the unbound drug concentrations [18,19].

The use of such tissue-composition-based equations to predict Vss has the advantage of providing a model with a clear interpretation about where drugs are being distributed; but it introduces the problem of obtaining the K_t:p_ coefficients for a number of tissues, for each drug. These are typically obtained via *in vivo* or *in vitro* experiments (whose limitations were briefly mentioned earlier) in animals, as they are difficult to be determined in humans [20]. Both *in vitro* and *in vivo* approaches are based on the measurement of drug concentration in the tissues and in the plasma at equilibrium or steady state. The model animals used in these studies are most often rat. In fact, rat is one of the most commonly used vertebrate in the estimation of pharmacokinetic parameters by interspecies scaling [21]. Due to the difficulty of obtaining experimental K_t:p_ values for a large number of drugs, an alternative approach consists of predicting K_t:p_ values and then using those predicted values to predict the Vss of a large number of compounds. This is essentially the approach that we propose here.

More precisely, we propose a new QSPkR approach consisting of two phases. In the first phase we obtained, from the literature, experimentally derived K_t:p_ values for a relatively small set of 110 compounds, and used that dataset to build QSPkR models predicting K_t:p_ values for different tissues based on molecular descriptors. In the second phase, we used the models built in the first phase to predict K_t:p_ for a larger set of 604 compounds, and then used the predicted K_t:p_ values as descriptors, in addition to molecular descriptors, to predict the Vss of compounds in that larger dataset. In this phase, we used the Vss dataset made available by Obach et al. [14]. This dataset has the advantages of being manually curated, being relatively large and containing only data collected from intravenous studies – which avoids the uncertainties about the degree of bioavailability in common routes of administration, like oral administration.

It is important to note that the dataset used here has a very diverse set of compounds, unlike other studies in the literature that focus on relatively small subsets of Obach et al.’s dataset – see, e.g., ref. [5,22]. On one hand, this large compound diversity makes it difficult to discover a model that predicts Vss with a high accuracy; but on the other hand the models produced in this study have a wider domain of applicability than other Vss models more specialized for certain classes of drugs. We will elaborate on this issue in the Discussion section.

The main contribution of this paper is to investigate whether or not the data mining-based prediction of the Vss of chemical compounds can be made more accurate by using as input not only the compounds’ molecular descriptors but also computationally predicted tissue:plasma partition coefficients (K_t:p_ values) for those compounds for a (sub) set of tissues. A secondary contribution of this work is that we discuss the pros and cons of several different types of decision tree-based regression methods and report the results of experiments comparing their predictive accuracy when building models to predict K_t:p_ and Vss. To our knowledge, these two contributions have not been reported yet in the pharmacokinetics or QSAR literature.

## Methods

The main dataset used in this research is a dataset containing 604 compounds with Vss values at steady state in humans, obtained from Obach et al.’s work [14], and our ultimate goal is to build models that predict human Vss for compounds in that dataset based on molecular descriptors and tissue:plasma partition coefficient (K_t:p_) descriptors, as mentioned in the Introduction. However, the experimental K_t:p_ values for different human tissues are unknown for the large majority of those compounds. Hence, in this study we propose a two-phase approach for building models predicting human Vss in steady state. In the first phase we build QSPkR models for predicting log K_t:p_ values for different tissues based on molecular descriptors; whilst in the second phase we used the predicted log K_t:p_ values as descriptors, in addition to molecular descriptors, to build QSPkR models predicting log Vss. These two phases are described in more detail next.

### The first phase of the proposed approach – building QSPkR models for predicting Log K_t:p_

**Figure 1:**
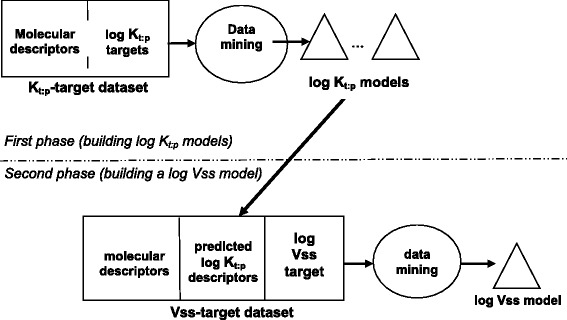
**Graphical summary of our two-phase approach for predicting log Vss.** First, 13 log K_t:p_’s (one for each tissue) are the target variables to be predicted from molecular descriptors. Then, the predicted log K_t:p_ values from these models together with molecular descriptors are used as descriptors to build models predicting log Vss.

Experimentally derived K_t:p_ values were available in the literature for a number of rat tissues for 110 compounds (see Additional file 1 for the dataset, including the original references for the data). ACDlabs logD 12.0 and MOE (Molecular Operating Environment) 2011 software were used to calculate about 300 molecular descriptors for each of these compounds, as described in detail in ref. [23]. This dataset was created with rat tissue K_t:p_, rather than human tissue K_t:p_, because there is substantially more data for the former than for the latter in the literature. Hereafter, we refer to that created dataset as the K_t:p_-target dataset. In essence, it included several types of drugs, such as NSAIDs, anticonvulsants, sulfonylureas, benzodiazepines, beta blockers, antipsychotics and some antibiotics such as cephalosporins, fluoroquinolones, tetracyclines, etc. Then, we applied data mining methods (described later) to the K_t:p_-target dataset in order to build a regression-type model for predicting the log K_t:p_ (where the log is in base 10) for each of 13 different tissues whose data is included in the Additional file 1, based on the molecular descriptors of the compounds. This process is summarized in graphical form in the top half of Figure 1.

Note that, for most compounds in the created dataset, there is a large proportion of missing values for the log K_t:p_ of most tissues (See Additional file 1). For one of the tissues, namely pancreas, only 3 compounds in the dataset have a known log K_t:p_ value, and so no attempt was made to build a regression model predicting pancreas log K_t:p_ values from so few compounds. For each of the other tissues, we tried to build regression-type models predicting the log K_t:p_ value for that tissue. This generated 13 different regression-type problems, each characterized by a different target (dependent, or response) variable, representing the log K_t:p_ of a different tissue, and a different (but overlapping) set of compounds. On the other hand, the set of descriptors (features, or independent variables) used to build the models was the same in all 13 regression problems, consisting of about 300 molecular descriptors.

In each of those regression-type problems, all compounds with known log K_t:p_ value for the target tissue were used to build and validate models predicting the target tissue’s log K_t:p_ value. For each tissue, we built several QSPkR models predicting that tissue’s log K_t:p_ value using different types of decision tree-based regression methods (described later). The error rate associated with each method was measured by a 10-fold cross-validation procedure, which works as follows [24]. First, the K_t:p_-target dataset was randomly divided into 10 folds. Next, the regression method was run 10 times, each time using a different fold as the validation set and the other 9 folds as the training set. The mean absolute error (MAE) on the validation set is computed for each run, and then averaged over the 10 runs and returned as the MAE for that method. Finally, the best model predicting log K_t:p_ for each tissue is chosen as the model produced by the method with the lowest MAE. The best model built for each tissue can then be used to predict that tissue’s log K_t:p_ value in the Vss-target dataset. Note that the use of a cross-validation procedure is not shown in Figure 1 in order to keep the figure simple.

It is important to note that, when building a model to predict a certain tissue’s log K_t:p_, the log K_t:p_s of other tissues are *not* used as descriptors. This restriction was implemented because a model built from the K_t:p_-target dataset is used later to predict a tissue’s log K_t:p_ values in the Obach et al.’s dataset, where other tissues’ log K_t:p_ values are unknown.

### The second phase of the proposed approach – building QSPkR models for predicting Log Vss

In this phase we used a larger set of 604 compounds and corresponding steady state Vss values in humans obtained from ref. [14]. The dataset published by Obach et al. consists of Vss values for 665 compounds. In this study, some of the compounds were removed because their molecular descriptors could not be calculated. This was the case for compounds containing metals, or other salts, or permanently charged compounds such as quaternary ammoniums. We also removed from the initial dataset all compounds that were included in our K_t:p_-target dataset. As a result, the final version of the dataset used in our experiments has 604 compounds. We refer to this dataset as the Vss-target dataset hereafter. As mentioned earlier, the log K_t:p_ values of different tissues are unknown for the large majority of compounds in the Vss-target dataset. Hence, we used the best regression models built in the first phase (i.e. the best model for each tissue) to predict the set of tissues’ log K_t:p_ values for the compounds in this dataset. Again, ACDlabs logD 12.0 and MOE (Molecular Operating Environment) 2011 software were used to calculate about 300 molecular descriptors for each compound in the Vss-target dataset [23]. Then we applied data mining methods to the Vss-target dataset in order to build regression models predicting log Vss in steady state in humans (where the log is in base 10), using as descriptors both the set of calculated molecular descriptors and the predicted log K_t:p_ values. We built models predicting log Vss, rather than Vss, because Vss has a very skewed distribution, with relatively few compounds having very high Vss values. This process is summarized in graphical form in the bottom half of Figure 1. In both phases, the regression models are expressed in the form of decision trees, as will be explained next.

### An overview of the decision tree-based regression methods used in this work

The type of regression method used in both the previously described phases of the proposed approach was decision-tree building algorithms. In essence, this type of method builds a decision tree by recursively partitioning the set of compounds in the training set, as follows. First, it considers all training set compounds, selects the descriptor that is the best predictor of the value of the target variable (a tissue’s log K_t:p_ in the first phase or log Vss in the second phase, in this work), adds the selected descriptor to the tree (as its root node), and partitions the training set based on the values of the selected descriptor. In this context, the best predictor is the predictor that partitions the data in a way that each of the resulting nodes has the minimum possible variance in the values of the target variable. Typically, in the case of numerical variables, this involves creating two training set partitions, one with compounds satisfying the condition *d*_*sel*_ ≤ *t* and the other with compounds satisfying *d*_*sel*_ > *t*, where *d*_*sel*_ is the selected descriptor and *t* is a threshold automatically chosen by the algorithm. Next, the same process of selecting the best descriptor, adding it to the tree and further partitioning the current set of compounds is applied to each of the just-created partitions (nodes). This process is recursively applied until a stopping criterion is satisfied for each partition, e.g. when the variance of the value of the target variable for the compounds in the current partition is below a pre-defined threshold, in which case a leaf node (terminal node) is created for that partition. The result of this process is a decision tree, where internal (non-leaf) nodes contain names of descriptors, the edges coming out from a node contain conditions like *d*_*sel*_ ≤ *t* or *d*_*sel*_ > *t*, and each leaf node predicts the value of the target variable for the compounds that have the descriptor values associated with the edges in the path from the root node to that leaf node. The leaf node’s prediction can be performed in different ways, associated with different types of decision trees, as discussed later.

We chose this paradigm of decision tree-based regression methods for several reasons. First, they produce graphical models that can be potentially comprehensible and interpretable by users [24-27]. This is in contrast to methods such as support vector machines and artificial neural networks, which produce models that are a kind of “black box”, being hardly interpretable by users. Second, they produce models capturing non-linear relationships in the data, instead of simply modeling only linear relationships, like traditional linear regression models often used in QSPkR studies. In addition, the paradigm of decision tree methods is broad enough to include several different types of decision trees (with different types of structures) for regression, which gives us more opportunities to try to find the best type of tree structure for our target regression problem. More precisely, in this work we compare the effectiveness of several types of decision tree-based model structures for regression, as follows.

#### (Conventional) regression trees

This type of decision tree structure has been popularized by the well-known CART (Classification and Regression Tree) algorithm [28]. In a regression tree, in each leaf (terminal) node, the log Vss value predicted for a new compound reaching that node is given by the mean of log Vss values over all the training set compounds that belong to that node. The main advantages of such regression trees are their simplicity and easy interpretation; i.e., each leaf node directly provides a predicted log Vss value, unlike the case of model trees, discussed next.

#### Model trees

This is a more sophisticated type of decision tree for regression. In a model tree, in each leaf node, the log Vss value predicted for a new compound reaching that node is given by a multivariate linear regression model built from the training set compounds that belong to that node [29]. Note that, at each leaf node, the linear model contains only descriptors that occur in tree nodes in the path from the root to the current leaf node or descriptors that occur in linear models somewhere in the subtree containing the current leaf node. After building such a linear model using standard linear regression techniques, the linear model can be simplified by removing irrelevant variables, using a greedy search procedure that tries to improve the model’s estimated error rate. Hence, a model tree performs embedded feature selection at two levels, i.e., at the level of internal (non-leaf) nodes and at the level of leaf nodes. The model tree approach is much more flexible than the conventional linear regression approach of building a single (global) linear model from the entire training set, because the latter makes the strong assumption that all the compounds have the same relationship between features and log Vss. By contrast, a model tree recognizes that different linear equations might describe well the relationship between features and log Vss for different subsets of compounds. Hence, in theory model trees can better adapt their structure to a diverse set of compounds, such as the Vss-target dataset used in this work. On the other hand, model trees tend to be more difficult to interpret than regression trees, since a single model tree can have a large number of linear equations (each with tens of descriptors) in its leaves. We will discuss the interpretation of model trees later.

#### If-then regression rules

A type of model conceptually similar to regression trees is a set of *If-Then* regression rules. Each rule has an “*If* part”, which consists of a set of conditions on the values of selected descriptors, and a “*Then* part”, which predicts the target variable’s value for any compound whose descriptors satisfy the conditions in the *If* part. Note that the *If* part is conceptually equivalent to a path from the root to a leaf node in a regression tree, and the *Then* part is conceptually equivalent to a leaf node. Actually, the method used to build *If-Then* regression rules in this work is based on decision trees [30]. In essence, it is an iterative method that, at each iteration, builds a decision tree, selects the “best” leaf node (the one with the largest estimated accuracy) and creates a rule corresponding to the path from the root to that leaf node, throwing away the rest of the tree. By iteratively repeating this procedure, it builds a set of modular *If-Then* rules, rather than a decision tree.

#### Bagging

Bagging (which stands for Bootstrap Aggregation) consists of an ensemble (or set) of decision trees, where different decision trees in the ensemble are produced by different random samplings (different bootstrap samples) of the original training set [31]. When we need to predict the log K_t:p_ or log Vss of a new compound, a predicted value is computed by each tree in the ensemble, and the predictions are averaged to give the ensemble’s prediction. Bagging can be used to produce a set of regression or model trees. In this work, all the trees in the ensemble produced by Bagging are model trees, since this type of decision tree produced somewhat better results than regression trees in our preliminary experiments. Bagging has the advantage of increasing the robustness of the model (reducing the variance of its predictions), by comparison with using a single decision tree, since its prediction is an average of the prediction of many models. However, Bagging has the disadvantage of producing a more complex model, which is considerably more difficult to interpret than a single decision tree. We will also discuss the interpretation of a Bagging model later.

#### Correlation-based feature selection (CFS) with genetic search

From a feature selection perspective, all the previously described decision tree-based regression methods perform “embedded feature selection”, in the sense that a decision tree is built by selecting the “best” feature to be used in each node of the tree, and normally only a proper subset of the input features is selected to occur in some tree node. A different type of feature selection method performs feature selection in a preprocessing phase, before running the decision tree building algorithm – or any other type of classification algorithm for that matter. It is often possible to achieve better predictive accuracy using a two-stage approach, where we first use a preprocessing feature selection method and then apply a decision tree building algorithm to the features selected in the first stage. For evidence that this approach can improve predictive accuracy over using just a decision tree building method, see e.g. Newby et al.’s work [32]. Hence, in this work we also tried to use one type of preprocessing feature selection method, namely genetic search-based CFS, which was also successfully used by Newby et al.. In essence, this method uses a genetic algorithm as a search method in the space of candidate feature (descriptor) subsets, and evaluates the quality of each candidate feature subset using a “fitness” (evaluation) function that considers two criteria: the correlation between features in that candidate subset and the target variable, and the redundancy among features in that candidate subset. The genetic search is used to find a feature subset that maximizes that correlation and minimizes that redundancy. For a review of the CFS method, see ref. [33], and for a review of genetic search applied to feature selection, see e.g. ref. [34].

## Results

### Results for the regression models predicting each Tissue’s Log K_t:p_ values

Table 1 shows, for each tissue, the mean absolute error (calculated by 10-fold cross-validation [24]) in the prediction of log K_t:p_ by each regression method, in our K_t:p_-target dataset. As discussed in the Methods section, we investigated the use of different types of decision tree-based regression methods. More precisely, the first four regression methods in Table 1 are variations of *model trees* built by the M5P algorithm in WEKA [29], where each variation used a different value of the parameter ‘minNumInstances’ (the minimum number of instances (compounds) to allow at a leaf node), namely the value 4, 6, 8 or 10, as indicated in the column headings. The column heading M5P-RegTree refers to the M5P version building *regression trees*, rather than model trees. REP-Tree is a method that produces a regression tree and was designed particularly to be fast, which may however lead to some reduction in its predictive accuracy, by comparison with other decision tree methods. M5-Rules is an M5P version that produces a model consisting of a list of *If-Then* regression rules, rather than regression trees [30]. The Bagging-M5P method [31] produces a set of M5P model trees and predicts, for a new compound, the value of a target variable that is an average of the values predicted by the individual model trees. The number of trees produced is a parameter, for which we used the default value of 10 in WEKA.

**Table 1:** **Mean Absolute Error (calculated by 10-fold cross-validation) in the prediction of log K**_**t:p**_ **value by different decision tree-based regression methods, for each tissue, in K**_**t:p**_ **-target dataset (with 110 compounds)**

**Tissue**	**M5P-4**	**M5P-6**	**M5P-8**	**M5P-10**	**M5P-RegTree**	**REP-Tree**	**M5-Rules**	**Bagging (M5P)**
muscle	0.3029	0.3040	0.3164	**0.2950**	0.3384	0.3857	0.3172	0.3228
brain	0.4695	0.4797	0.4869	0.4768	0.5387	0.5347	0.5170	**0.4613**
intestine	0.5939	0.4700	0.5337	0.5245	**0.4523**	0.4698	0.6060	0.5361
lung	**0.4549**	0.4812	0.4755	0.4776	0.5710	0.5522	0.5114	0.4822
spleen	0.9824	1.0069	1.0276	1.0206	0.8149	0.7997	0.8783	**0.7857**
heart	0.3391	0.3076	**0.2991**	0.3321	0.3562	0.4116	0.3723	0.3358
skin	0.2844	0.2752	0.2954	**0.2666**	0.3436	0.3433	0.2782	0.2961
bone	0.6210	0.7101	0.5893	0.5735	0.5109	0.5847	0.5724	**0.4762**
adipose	0.3581	0.4106	0.4186	0.4005	0.4557	0.5268	0.4031	**0.3214**
kidneys	0.2848	0.2706	0.2756	0.2736	0.3226	0.4035	0.2913	**0.2281**
liver	0.5495	0.5634	0.5036	0.5091	0.4925	0.5569	0.5879	**0.4608**
gut	0.6484	0.6134	0.6056	0.4905	0.4695	0.3912	0.4135	**0.3169**
thymus	0.3943	0.3599	0.3667	0.3601	0.2633	**0.2176**	0.3529	0.2623

In Table 1, for each tissue, the smallest Mean Absolute Error (MAE) – among the 8 regression methods – is highlighted in bold. As expected, there is no single regression method that is the best across all tissues, but overall the Bagging-M5P method is the most successful one. It obtains the smallest MAE in 7 out of the 13 tissues. Recall, however, that our goal is not to select the best regression method overall, but rather to select the best regression method to predict the log K_t:p_ value for each tissue. The best model built for each tissue was then used to predict that tissue’s log K_t:p_ value for all compounds in the Vss-target dataset, where those predicted log K_t:p_ values are used as descriptors (in addition to a large number of molecular descriptors) to build and validate models predicting log Vss.

As can be seen in Table 1, the value of the best (smallest) MAE for each tissue varies from 0.2176 and 0.2281 for the log K_t:p_’s of thymus and kidneys, respectively, to 0.7857 for the log K_t:p_ of spleen. Given this significant variability in the MAE values, one could wonder whether just a subset of the models having relatively small MAEs (e.g., values below a certain threshold) should be used for predicting the tissue log K_t:p_ values to be used as descriptors in the Vss-target dataset, whilst other tissues’ log K_t:p_ values (with large MAEs) should be ignored. However, this would introduce the problem of how to determine which MAE values are small enough for their corresponding predicted log K_t:p_ to be reliably used as descriptors in the Vss-target dataset. A predefined threshold would be an ad-hoc solution. In addition, the MAE value by itself seems not to be an effective measure of the usefulness of a predicted log K_t:p_ value, because the predicted values will be used as descriptors for building models predicting log Vss in the Vss-target dataset, which is a regression problem very different from the problem of building the models reported in Table 1.

Hence, once the best model predicting log K_t:p_ has been selected for each tissue based on the results shown in Table 1, the decision about which of those models will be used as descriptors should be made by directly taking into account the predictive performance of each predicted log K_t:p_ descriptor in log Vss models. In our case, this is naturally done by taking advantage of the fact that the decision tree-based regression methods used to predict log Vss perform an embedded ‘descriptor selection’ (or feature selection) procedure as part of the decision tree building process – recall that only descriptors considered relevant for predicting the target variable are included in the decision tree. That is, we let the decision tree-based regression methods use, as input descriptors, the set of predicted log K_t:p_ values for (almost) all the tissues – in addition to the aforementioned large set of molecular descriptors. Then the tree-building algorithm automatically decides which of those predicted log K_t:p_ descriptors – as well as which molecular descriptors – are relevant enough to be included in the decision tree predicting log Vss. Out of the 13 tissues shown in Table 1, there is only one whose best log K_t:p_-predicting model was not used to fill in the corresponding descriptor values in the Vss-target dataset, namely the model for intestine. This is because the best model for this tissue’s log K_t:p_ consists of a degenerated decision tree having only one leaf node, which predicts the same value of intestine log K_t:p_ for all new compounds, making it useless as a descriptor.

### Results for the regression models predicting Log Vss

In order to evaluate the predictive performance of models predicting log Vss, the Vss-target dataset was randomly divided into two sets. One set, with 402 compounds, is used as the model selection set; whilst the other set, with the remaining 202 compounds, is used as an external validation set. To perform model selection, each of the aforementioned decision tree-based regression methods was applied to the model selection set, using 10-fold cross-validation. Then the best model – i.e., the one with smallest mean absolute error (MAE) – is selected, and that model is used to predict log Vss for the compounds in the external set. We emphasize that the compounds in the external set were not used in the model selection set (nor in the K_t:p_-target dataset), i.e., the external set compounds were not used in any way to build or select models, and so the predictive performance in the external set represents a fair measure of the generalization ability of the used decision tree-based regression models.

Table 2 shows the MAE – calculated by 10-fold cross-validation – in the prediction of log Vss in the model selection dataset. This table has 3 columns with results. The first one reports results for each regression method when using, as input descriptors, both the set of 12 tissue log K_t:p_s whose values were predicted by the corresponding best model in Table 1 and a set of about 300 molecular descriptors – as explained earlier. The next column reports the results for each regression method when using, as input descriptors, only the set of molecular descriptors. Hence, in those experiments the predicted tissue log K_t:p_s were not used as descriptors for predicting log Vss, providing us with a baseline set of experiments against which we can measure the influence of using predicted tissue log K_t:p_s as descriptors.

**Table 2:** **Mean Absolute Error (MAE) – calculated by 10-fold cross-validation – in the prediction of log Vss by each regression method, in model selection dataset (with 402 compounds)**

**Regression method**	**MAE (logVss)**		
	**Descriptor set includes predicted log K**_**t:p**_ **’s**	**Descriptor set without predicted log K**_**t:p**_ **’s**	**Descriptor set selected by genetic CFS**
M5P-4	0.3891	0.3698	0.3739
M5P-6	0.3823	0.3665	0.4003
M5P-8	0.4715	0.3616	0.3847
M5P-10	0.4751	0.3658	0.3763
M5P-RegTree	**0.3689**	0.3772	0.3782
REPTree	0.3824	0.4330	0.3974
M5Rules	0.3911	0.3843	0.3836
Bagging (M5P)	0.3713	**0.3371**	**0.3509**

The last column in Table 2 reports the results of another set of experiments involving a two-step feature selection approach, as follows. First we apply a feature (descriptor) selection method to the dataset containing, as input descriptors, both the set of 12 tissue log K_t:p_s whose values were predicted by the corresponding best model in Table 1 and the large set of molecular descriptors. The feature selection method used was Correlation-based Feature Selection (CFS) with genetic search (see Methods section). Out of the 56 descriptors selected by the CFS method, only two are predicted log K_t:p_ descriptors, namely the log K_t:p_ for adipose tissue and thymus. In the second step, the 56 descriptors selected by CFS are used as input by a decision tree-based regression method. Note that in the first step a descriptor subset is selected by CFS in a preprocessing phase, independent from the regression method; and in the second step the embedded descriptor selection procedure of the regression method further selects a (usually smaller) subset of relevant descriptors. This kind of two-step feature selection approach has also been successfully used to build other types of QSPkR models [32].

In Table 2, the best result (smallest MAE) for each type of descriptor set is highlighted in bold. As can be observed in the Table, the M5P algorithm generating a regression tree produced the best model when using the first type of descriptor set, whilst Bagging produced the best models when using the other two types of descriptor sets.

Finally, for each of the three types of descriptor sets, the best model identified in Table 2 was used to predict the log Vss of all compounds in the external set. The results are shown in Table 3. The Geometric Mean Fold Errors (GMFEs) shown in that table were calculated as: GMFE = antilog_10_ (MAE) [3]. The GMFE measure has the advantage of being less affected by extreme outliers, by comparison with the coefficient of determination (which measures the quadratic error) [35]. In order to interpret the GMFE measure, note that a model with a GMFE of 2 makes Vss predictions which are on average twofold off – i.e., 100% above or 50% below the true Vss value.

**Table 3:** **Mean Absolute Error (MAE) and Geometric Mean Fold Error (GMFE) calculated for each combination of input descriptor set and the best regression model for that descriptor set, when predicting log Vss for all compounds in the external set (with 202 compounds)**

**Model**	**Input descriptor set**	**Regression model type**	**MAE**	**GMFE**
1	predicted log K_t:p_’s and molecular descriptors	M5P – regression tree	0.4172	2.61
2	molecular descriptors only (i.e., without predicted log K_t:p_’s)	Bagging (a set of M5P model trees)	0.3676	2.33
3	descriptor set selected by genetic search-based CFS	Bagging (a set of M5P model trees)	0.3609	2.29

As can be observed in Table 3, the approach of using as descriptors both the predicted log K_t:p_ values for 12 tissues and a large set of molecular descriptors (model 1) was not successful, by comparison with the baseline approach of using as descriptors only a large set of molecular descriptors (model 2). The former approach obtained a GMFE of 2.61, versus a GMFE of 2.33 for the baseline approach. However, when the more sophisticated approach of selecting descriptors with both the genetic search-based CFS and the decision tree method was used (an approach that also used predicted log K_t:p_ values as descriptors), a small improvement over the baseline approach was obtained, with the GMFE being slightly reduced from 2.33 to 2.29. A *T*-Test of mean difference with a 95% confidence interval indicated the difference between the prediction errors was not statistically significant (confidence interval of (−0.031, 0.017) and p = 0.577). Despite this, the significance of log K_t:p_ in the prediction of Vss was very clear based on the fact that all the models where log K_t:p_ parameters were included as input parameters (Model 1 and all the model trees making up Model 3) used a log K_t:p_ parameter as the most important descriptor (see model interpretations in the next two sections).

The fact that the approach of using the predicted log K_t:p_ values as descriptors without running genetic search-based CFS (model 1) led to a higher GMFE than the baseline may be due to a number of factors. One explanation may be the two phase approach adding a level of uncertainty due to the use of predicted, as opposed to the experimentally measured, K_t:p_ values. Despite the advantages of using tissue distribution from a mechanistic point of view, the use of predicted tissue to plasma partition coefficients can subject the model to the errors associated with K_t:p_ prediction. This is in addition to the experimental error associated with the *in vitro* or *in vivo* measurements of partition coefficients [36], which is especially variable for basic lipophilic compounds [37]. Therefore, it is essential to ascertain the quality of predicted K_t:p_ values for the compounds in Vss-target data set. It is usually noted that prediction by a QSAR model is reliable only to compounds that are similar to the training set compounds [38]. In this case, the compounds in the Vss-target dataset need to be within the molecular descriptor boundaries of the K_t:p_ dataset.

Here, we used a descriptor range-based approach to identify compounds within or outside the molecular descriptor space. Since only one log K_t:p_ parameter has been used in the set of model trees built by M5P in model 3 in Table 3, namely K_t:p_ for the adipose tissue, the descriptor boundary was investigated only for this parameter. First, the set of descriptors that was used for the prediction of adipose tissue’s log K_t:p_ were identified. This set contains 16 descriptors. Then, compounds in the Vss validation set that have at least one descriptor value outside the descriptor values of the K_t:p_ dataset were identified. Out of 202 compounds in the Vss validation set, 43 compounds (21%) were identified as falling outside the range of the descriptor values of the K_t:p_ dataset. The average error for all validation set compounds and the validation set excluding the 43 compounds were found to be similar (2.29 for all compounds vs 2.34 for compounds that are within the descriptor space). The reason for a very similar error for the validation set compounds within and those outside the descriptor range could be due to the source of Vss error being other parameters than the uncertainties of log K_t:p_ prediction. For example, one important observation for the compounds showing high Vss prediction error (by all methods) is that the large majority of these compounds have a relatively extreme (very high or very low) Vss value, which seems to be the main reason for their large errors. In other words, these major outliers are “prediction outliers” rather than “descriptor outliers”. As further evidence, a comparison of the compounds that are outside the descriptor boundary with those that are within the descriptor range shows that in general compounds with similar chemical and pharmacological nature can be found in both groups. For example, three out of nine cephalosporins and one out of seven penicillins and two out of four quaternary ammonium muscle relaxants are outside the domain with the remaining similar structures within the boundary.

To further evaluate the reliability of the predicted log K_t:p_ for adipose tissue for the prediction of Vss, we performed a sensitivity analysis to investigate how uncertainty in the regression for adipose tissue’s log K_t:p_ prediction affects the prediction of Vss. More precisely, we performed a controlled experiment where we artificially introduced 10% of noise to the predicted adipose tissue’s log K_t:p_, as follows. For each compound in the entire Vss-target dataset (including both the training (model selection) and external datasets), the value of the predicted adipose tissue’s log K_t:p_ descriptor in that compound was modified by adding or subtracting 10% of the current descriptor value, where the decision to perform addition or subtraction was made at random. This procedure was repeated five times, varying the random seed used to decide if the 10% of noise was added or subtracted to each compound, which led to 5 new modified versions of the Vss-target dataset. For each of these 5 modified Vss-target datasets, we ran again the Bagging M5P regression algorithm and measured its geometric mean fold error (GMFE), averaging the results over the 5 runs. This procedure led to a GMFE or 2.35, which should be compared to the GMFE of 2.29 obtained by Bagging using the original predicted adipose tissue’s log K_t:p_ descriptor values (without any extra artificial noise) – as reported in Table 3. Hence, a small degree of noise added to the predicted adipose tissue’s log K_t:p_ only moderately affected the prediction of Vss, giving us more confidence in the relevance of this tissue’s log K_t:p_ to predict Vss.

In order to further understand the effect of this small degree of noise in the predictive power of the predicted adipose tissue’s log K_t:p_ descriptor, we also computed the frequency of occurrence of this descriptor in the root node of the model trees built by Bagging M5P. Recall that a model tree’s root node contains the most relevant descriptor for predicting Vss. When using the original predicted adipose tissue’s log K_t:p_ descriptor values (without any extra artificial noise), that descriptor occurs as the root node in all the 10 model trees built by Bagging. On the other hand, using the predicted adipose tissue’s log K_t:p_ descriptor values with 10% of noise, this descriptor occurs as the root node, on average, in 8.6 of the 10 model trees. So, again the decrease in the relevance of this descriptor was not great; it is still the most relevant descriptor overall in the set of model trees built by Bagging, even with 10% noise added to its value, which further reinforces our confidence in the predictive power of this descriptor.

Distribution coefficients are the main factors controlling the Vss of drugs. However, here we used data for rat K_t:p_, rather than human K_t:p_, when building models predicting log K_t:p_. As mentioned earlier, this was due to the availability of more rat K_t:p_ data than human K_t:p_ data in the literature, but clearly there are species differences that lead to different values of K_t:p_ for rats and humans [6,39]. In addition, even when using rat K_t:p_ data, the number of compounds with known log K_t:p_ value available for some tissues (i.e. data required to build the model for those tissues) was still relatively small, which also limited the predictive accuracy that could be achieved by the log K_t:p_ models. However, the fact that the third approach in Table 3, selecting descriptors (including predicted log K_t:p_ values) using both the genetic search-based CFS and the decision tree method, achieved the best results overall suggests that at least some tissue(s)’ log K_t:p_(s) were predicted well enough and were correlated with Vss strongly enough to improve the predictive accuracy, by comparison with the baseline approach of not using predicted log K_t:p_ values.

Hence, a natural question to ask is whether the descriptors representing predicted log K_t:p_ values are often selected to be included in the decision tree models, in the case of the first and third approaches in Table 3. This question is discussed in the next two sections. Note that the question is not valid in the case of the second approach, where predicted log K_t:p_ values are not used as descriptors.

### Interpreting the regression tree for predicting Vss built by M5P from predicted K_t:p_ and molecular descriptors

The best model built by M5P when using as input the 12 predicted log K_t:p_ descriptors for different tissues is a regression tree where log K_skin:plasma_ is the most relevant descriptor, occurring at the tree’s root node. Note that, due to its position at the root of the tree, log K_skin:plasma_ will be used to predict the log Vss of every new compound, since the root node is included in all the paths leading to all the leaf nodes in the tree. The regression tree also contained log K_muscle:plasma_, but this occurred at a deeper position in the tree, and therefore it is used to predict the log Vss of a much smaller number of compounds, by comparison with the log K_t:p_ for skin. The entire regression tree has 34 nodes (16 internal nodes and 18 leaf nodes), which is too large to be visualized here. Hence, instead of showing the regression tree, we show here a subset of the *If-Then* rules extracted from that tree. Recall that each path from the root to a leaf node in a regression tree is equivalent to an *If-Then* rule where the antecedent (“*If* part”) contains the conditions associated with values of the descriptors in the internal nodes and the consequent (“*Then* part”) contains the log Vss value predicted for any compound that satisfies the conditions in the rule’s antecedent. Analyzing a list of rules extracted from a tree, rather than directly analyzing the tree, helps us to interpret the model in a more modular way [26,27], since each rule can be interpreted independent from the others, unlike the entanglement of paths in a decision tree. Hence, this approach for improving model interpretability is often used in data mining, particularly when the original tree is relatively large, which is the case in this work.

Table 4 shows the subset of rules extracted from the regression tree satisfying the criterion that each rule covers at least 10 compounds in the model selection dataset – where the coverage of a rule is the number of compounds satisfying the conditions in the antecedent of the rule. We focus on these rules because they can be considered more reliable, since the rules covering less than 10 compounds have less statistical support for their predicted log Vss value. Note that, although the regression tree predicts log Vss, rather than Vss, in order to facilitate the interpretation of the results the second column of Table 4 shows the actual Vss value in L/Kg.

**Table 4:** ***If-Then*** **regression rules with coverage ≥ 10 extracted from the regression tree built by M5P when using as input descriptors both the predicted log K**_**t:p**_ **for 12 tissues and a large set of molecular descriptors**

**Rule**	**IF (a set of conditions)**	**THEN Vss (L/Kg)**	**coverage**
1	log K_skin:plasma_ ≤ 0.044 *and* SlogP_VSA7 ≤ 88.002	0.57	80
2	log K_skin:plasma_ ≤ 0.044 *and* SlogP_VSA7 > 88.002 *and* ASA– ≤ 181.026	0.37	30
3	log K_skin:plasma_ ≤ 0.044 *and* SlogP_VSA7 > 88.002 *and* ASA– > 181.026	0.27	60
4	log K_skin:plasma_ > 0.044 *and* a_ICM < = 1.553 *and* fiA < = 0.017 *and* PEOE_VSA+0 < = 108.431 *and* PEOE_RPC– < = 0.187	1.66	13
5	log K_skin:plasma_ > 0.044 *and* a_ICM < = 1.553 *and* fiA < = 0.017 *and* PEOE_VSA+0 < = 108.431 *and* PEOE_RPC– > 0.187	1.96	26
6	log K_skin:plasma_ > 0.044 *and* a_ICM < = 1.553 *and* fiA < = 0.017 *and* PEOE_VSA+0 > 108.431 *and* SlogP_VSA4 < = 16.939	3.14	20
7	log K_skin:plasma_ > 0.044 *and* a_ICM < = 1.553 *and* fiA < = 0.017 *and* PEOE_VSA+0 > 108.431 *and* SlogP_VSA4 > 16.939	2.15	19
8	log K_skin:plasma_ > 0.044 *and* a_ICM > 1.553	1.10	100

Note that log K_skin:plasma_ occurs in all rules shown in Table 4 because it is the root node in the regression tree from which the rules were extracted, as mentioned earlier. The first three rules in the table are the only rules in the original tree containing the condition log K_skin:plasma_ ≤ 0.044, and all those rules predict small Vss values, considerably below 1 L/Kg. These are the only rules predicting Vss < 1 L/Kg out of the entire set of 18 rules corresponding to the 18 leaf nodes in the original tree, i.e., all rules containing the condition log K_skin:plasma_ > 0.044 predict Vss values higher than 1. The other descriptors SlogP_VSA7 and ASA– are used in the first three rules to refine the predicted Vss value; in particular the condition SlogP_VSA7 > 88.002 is associated with even smaller Vss values. SlogP_VSA7 is the sum of accessible van der Waals surface area for atoms whose atomic contribution to logP(o/w) is in (0.25,0.30). These are specific atom types that include acidic hydrogens and aromatic and non-aromatic carbon atoms in certain positions, e.g. attached to other aromatic groups (see ref. [40] for a full description of these atoms). The effect of SlogP_VSA7 on Vss values indicates that drugs with large resonance area (e.g. more than one aromatic ring) that contain acidic groups such as COOH have low Vss. This is in agreement with previous literature, as acidic drugs are known to have low Vss values in general [3,41]. Furthermore, it can be seen from Table 4 that out of these acidic and aromatic compounds, those with large surface area of negatively partially charged atoms (ASA-) have even lower Vss values. Large ASA- indicates compounds with many electronegative atoms, a parameter similar to polar surface area that is known to have a negative effect in membrane penetration [42,43].

Turning to the rules with log K_skin:plasma_ > 0.044 in Table 4, the first four ones (i.e. rules 4–7) are somewhat more difficult to be interpreted, since they have five conditions in their antecedent, but they all have three conditions in common in their antecedents: “log K_skin:plasma_ > 0.044 *and* a_ICM < = 1.553 *and* fiA < = 0.017”, where a_ICM is atom information content and more precisely the entropy of the element distribution in the molecule, and fiA is the fraction of molecules that are ionized as acid at physiologic pH. This part of the rule indicates that compounds with high ability to partition into skin that also contain fewer heteroatoms and are not considerably acidic have relatively high Vss values, according to rules 4–7 in Table 4. Given those three conditions, those rules’ predictions depend on the value of PEOE_VSA+0 and another descriptor. PEOE_VSA+0 is sum of van der Waals surface area of atoms that have a close to neutral partial atomic charge (in the range (0.00,0.05) with PEOE charge calculation [44]). For compounds that satisfy the above three conditions on log K_skin:plasma_, a_ICM and fiA, broadly speaking the condition PEOE_VSA+0 < = 108.431 is associated with smaller Vss values than the condition PEOE_VSA+0 > 108.431, as can be seen by comparing the 4th and 5th rules in Table 4 against the 6th and 7th rules. This indicates higher Vss values for molecules containing mostly neutral (or less polar) atoms. In the case of the two rules with PEOE_VSA+0 < = 108.431, the use of the descriptor PEOE_RPC– in the last condition to refine the rules does not have much impact on the predicted Vss. However, in the case of the two rules with PEOE_VSA+0 > 108.431, the predicted value is significantly affected by the value of SlogP_VSA4, another SlogP-related descriptor. This is the sum of van der Waals surface area of specific atom types with logP(o/w) contribution in the range (0.1,0.15]. These atoms include aliphatic carbon and hydrogens and carbonyl groups attached to aromatic rings, which are more prevalent in larger molecules with many heteroatoms such as paclitaxel, cyclosporine and saquinavir. Consistently with the occurrence of SlogP_VSA7 in the first three rules shown in Table 4, a larger value of SlogP_VSA4 (in this case, > 16.939) is associated with a smaller predicted Vss, namely 2.15, versus 3.14 when SlogP_VSA4 < = 16.939. Finally, the last rule in Table 4 is “*If* log K_skin:plasma_ > 0.044 *and* a_ICM > 1.553 *Then* Vss = 1.10”. This rule predicts a Vss of 1.10, which is the lowest predicted Vss value among all 15 rules with condition log K_skin:plasma_ > 0.044 found in the original tree. The compounds with high a_ICM have a higher ratio of different heteroatoms in the molecule. It is also the most generic rule found in the original tree, with a coverage of 100 compounds that includes β-lactam and quinolone antibiotics, antivirals such as guanosine analogues and similar relatively polar compounds.

In summary, according to the regression tree model built by M5P, the most relevant descriptor for predicting log Vss is log K_skin:plasma_, where larger values of this descriptor are associated with higher log Vss values. In addition, other (molecular) descriptors can be used together with log K_skin:plasma_ to improve log Vss prediction. In particular, larger values of the related descriptors SlogP_VSA7 and SlogP_VSA4 along with heteroatom ratio (a_ICM) are associated with smaller predicted Vss values – in the context of the values of other descriptors occurring in the same rule antecedents as those two descriptors.

### Interpreting the model trees for predicting Vss built by bagging M5P from the descriptors selected by CFS

The best regression model built by M5P when using as input only the descriptors selected by the genetic search-based CFS method in a preprocessing phase is a Bagging model, consisting of 10 model trees. The interpretation of this regression model is complicated because in each model tree, at each leaf (terminal) node, there is a multiple linear regression model. Each such linear regression equation typically has about 30 descriptors. In addition, there are in total 244 such linear regression equations in the set of 10 model trees, making their interpretation very difficult in practice. Therefore, in terms of interpretability of the Bagging regression model, we focus mainly on identifying the most relevant descriptors occurring in the internal (non-leaf) nodes of the 10 model trees, rather than on the descriptors occurring in the more numerous linear models.

Identifying only the most relevant descriptors occurring in the internal nodes of a set of decision trees (produced, e.g., by Bagging or random forests) is actually relatively common in the literature, and it can be performed in different ways. One approach consists of computing the percentage of the number of times that each descriptor was selected as the split variable in a tree, out of the number of times the descriptor could be selected (i.e., the total number of nodes in all trees), and then report the top descriptors ranked according to that percentage of selection frequency. This approach was used e.g. in ref. [45], where the top 10 descriptors were reported. However, this approach implicitly assumes that the occurrence of a descriptor in a tree node has the same importance regardless of the level of that node in the tree. This assumption is far from true, because, broadly speaking, descriptors at shallow (close to the root) nodes are more relevant than descriptors at deep (far from the root) nodes. This is because, when using any decision tree for predicting the log Vss value of new compounds, each compound will be assigned to a single path in the tree from a root to a leaf node, and in general shallow nodes occur in many more paths than deep nodes. In particular, a descriptor at the root node will be used to classify every new compound, since it occurs in all paths from the root to any leaf node, as mentioned earlier. In contrast, a descriptor that occurs, say, twice in the tree at deep levels (say at the 4th and 5th levels) will be used to classify much fewer compounds, being therefore considerably less relevant (despite occurring twice in the tree) than the root descriptor.

Hence, taking into account that descriptors at shallow tree levels are in general more relevant than descriptors at deep tree levels, we report in Table 5 the descriptors selected by M5P to occur either at the root node or at one of the root’s child nodes, for the best model produced in our experiments when using as input only the descriptors selected by genetic search-based CFS method applied in a preprocessing step. The table shows only descriptors occurring at least twice as a root or its child in some model tree. We did not consider descriptors that were chosen in such roles just once because their occurrence is less reliable and may be due mainly to some stochastic effect of the Bagging method.

**Table 5:** **Most relevant descriptors occurring in the set of 10 model trees produced by Bagging M5P to predict log Vss when using as input only the descriptors selected by genetic search-based CFS**

**Descriptor**	**Frequency in root node**	**Frequency in child of root node**
log K_adipose_tissue:plasma_	10	0
PEOE_VSA+0	0	3
Log P	0	2
TPSA	0	2
C_ratio	0	2
Vsurf_ID6	0	2

In Table 5, it is interesting to note that, in all of the 10 model trees built by Bagging, the descriptor selected for the tree’s root node was log K_adipose_tissue:plasma_. Hence, this can be considered by far the most relevant descriptor (out of the descriptors pre-selected by the CFS method) as evaluated by the Bagging M5P algorithm. Note that no other tissue’s log K_t:p_ descriptor was found to be very relevant according to the criteria used to produce Table 5. In addition, it should be recalled that log K_adipose_tissue:plasma_ was selected by the CFS method in a preprocessing phase, unlike most other tissues’ log K_t:p_s. A possible explanation for the much greater importance of log K_adipose_tissue:plasma_, by comparison with the other tissues’ log K_t:p_s, is that the other tissues’ log K_t:p_ values are redundant with respect to other molecular descriptors selected by Bagging; whilst log K_adipose_tissue:plasma_ is not only strongly correlated with Vss, but is also non-redundant (or at least less redundant) with respect to the other molecular descriptors selected by Bagging. Indeed, it has been reported in the literature that adipose tissue has some special characteristics that may make it particularly distinguishable from other tissues, in the context of Vss prediction. More precisely, adipose tissue is fatty and has a higher percentage of neutral lipids, and in adipose tissue the distribution may be dominated by lipophilicity and hydrophobic interactions, rather than by electronic interactions like in other tissues [39]. In addition, in a recent study proposing a physiologically-based pharmacokinetic model for improving the prediction of tissue distribution and volume distribution of highly lipophilic compounds [19], the simulation of the partition coefficient for adipose tissue was considered more sensitive to lipophilicity, by comparison with a non-adipose tissue. Interestingly, another tissue with a more lipophilic composition is skin [39], and, in the regression tree built by M5P when using as input the 12 predicted log K_t:p_ descriptors for different tissues, the most relevant descriptor chosen for the root node was log K_skin:plasma_, as discussed in the previous section.

The next descriptor mentioned in Table 5 is PEOE_VSA+0 (sum of van der Waals surface area of the least polar atoms with low partial atomic charges), which occurred at a child of the root node in 3 out of the 10 model trees.

Another group of relevant descriptors reported in Table 5 includes log P (the log of n-octanol-water partition coefficient), TPSA (Topological Polar Surface Area), C_ratio (the ratio of the number of carbon atoms over the total number of atoms in the compound) and Vsurf_ID6 (Hydrophobic integy moment), each occurring twice as a child of the root node in the set of model trees. Out of those, log P is a very common measure of lipophilicity, but C_ratio can also indicate compounds’ lipophilicities, since higher carbon ratios indicate fewer polar heteroatoms. Log P and other lipophilicity descriptors are often considered relevant predictors of Vss in the literature, since in general increased values of log P lead to increased values of Vss [4,13,14,45]; although this general tendency may not be always true for compounds with extremely high log P values [19]. Also, log P and other lipophilicity descriptors are not identified as relevant in every study; an example exception is reported in [22], where the authors did not find any direct relationship between Vss and lipophilicity descriptors. However, in that study polarity descriptors often occurred in the Vss models, and the authors noted that polarity is inversely related to lipophilicity. In a recent study by Paixao et al., who incorporated eight input molecular descriptors in their artificial neural network model based on the literature studies on drug distribution, the relative importance of the descriptors was investigated by varying each input at a time and considering all the other descriptors as constant. This study identified similar parameters, including log P, TPSA and hydrophilicity index as the important determinants of drug distribution [46]. In our experiments, log P seems both to be a relevant descriptor of Vss and to have a positive correlation with Vss.

## Discussion

### Related work on building QSPkR models for predicting Vss using Obach et al.’s dataset

A direct comparison between the models produced in this work and other models predicting Vss reported in the literature is complicated by the differences in the datasets and types of regression methods used. Concerning dataset variations, there are several other studies based on Obach et al.’s dataset [14], including [5,22,35,45]. However, unlike our work, most of those studies tend to use a substantially smaller version of Obach et al.’s dataset, focusing on a single type of compounds or removing compounds that are more difficult to predict for some reason. In particular, the dataset used by Louis and Agrawal [5] contains drugs that belong to the category of anti-infective (J) and sub-categories antibacterial (J01), antimycotics (J02) antimycobacterial (J04) and antiviral (J05) according to the anatomical therapeutic classification (ATC). That dataset contains only 126 compounds, whose Vss varies from 0.05 to 33 L/Kg. The smaller diversity of compounds and their smaller Vss range helps to improve the predictive accuracy of the models, at the expense of a more narrow applicability domain [22,45].

Another study using just a relatively small subset of compounds from Obach et al.’s dataset is reported by Zhivkova and Doytchinova [22]. In that work the dataset consisted of a more heterogenous set of structurally diverse drugs, but the data used to build the models contained only 132 acidic drugs, with an external set containing only 10 acidic drugs that were compiled from ref. [47] and were not included in Obach et al.’s dataset. It is known that acidic drugs tend to have relatively small values of Vss, compared with basic drugs, due to the fact that acids tend to have extensive binding to plasma proteins [41]. Indeed, in the dataset used by Zhivkova and Doytchinova, the compounds’ Vss values range from 0.04 to 15 L/kg, an even smaller range than the one in the dataset used by Louis and Agrawal [5]. Again, the smaller Vss range in the dataset used by Zhivkova and Doytchinova helps to improve the predictive accuracy of the models, at the expense of the models’ applicability being restricted to acidic drugs.

In the work of Demir-Kavuk et al. [35], in order to avoid missing values for some descriptors, many compounds for which some descriptors could not be calculated were removed from the original Obach et al.’s dataset. More precisely, compounds containing phosphorous, boron and metal atoms, all macrocycles, and some fragment-like compounds (like Metformin) were removed, which reduced the size of the dataset to 584 compounds. By contrast, in this current work we did not remove any compounds due to missing descriptor values, since the regression methods in the WEKA data mining tool used in this work can cope with missing values (see ref. [24] for details); and this allowed us to work with a somewhat larger dataset of 604 compounds (the size of our Vss-target dataset after the removal of compounds that occurred in our K_t:p_-target dataset).

In the work of del Amo et al. [45] a larger subset of Obach et al.’s dataset was used, containing 642 drugs – even larger than our Vss-target dataset, since they did not need to remove compounds occurring in a separate K_t:p_-target dataset (they do not use any dataset equivalent to that). In that study idadronic, pamodronic, risedronic and zoledronic bisphosphonates were removed from the original dataset based on the argument that these compounds are sequestered to the bones, which hinders their detection in plasma and leads to underestimated Vss values. In addition, two antimalarial drugs, namely hydroxychloroquine and chloroquine, were also removed from the original dataset due to their very high Vss values of 700 L/Kg and 140 L/Kg, respectively, which are far from the range of Vss values of the other compounds in their dataset (from 0.035 to 60 L/Kg). The latter two drugs were included in our external set, which contributed to a larger prediction error – this issue is further discussed later, when we mention some outliers to our models’ prediction.

### Related work on decision tree-based regression methods for predicting Vss

Among the several aforementioned studies building QSPkR models from subsets of the Obach et al.’s dataset, the one using the most related data mining method is the work by del Amo et al. [45], where decision trees are used for predicting Vss. By contrast, the studies performed by Demir-Kavuk et al. [35], Louis and Agrawal [5], and Zhivkova and Doytchinova [22] focused mainly on using variations of multiple linear regression, without building decision tree models. Hence, in the following we initially focus on discussing the decision tree-based regression methods used by del Amo et al. [45], contrasting them with the decision tree-based methods used in this work. Next, we discuss other related work on using decision tree-based regression models for predicting Vss.

First of all, note that in del Amo et al.’s work the decision trees are used to predict discrete classes of Vss value, namely classification into ‘high’, ‘medium’ or ‘low’ Vss groups. In contrast, in our work the decision trees perform regression, predicting the numerical value of log Vss.

Another work using decision tree-based methods to predict Vss is reported by Lombardo et al. [13]. The main type of QSPkR model discussed in that study was a hybrid mixture discriminant analysis (MDA)/random forest model. The MDA model is built to discriminate between high or low Vss values, which are defined as above or below the threshold of 10 L/Kg. In addition, two different random forest models are built for predicting the numerical values of high and low Vss compounds. The prediction of Vss for a new compound is then performed in two stages. Firstly, the MDA model predicts just whether a compound has a high or low Vss. Secondly, the numerical value of Vss is predicted by the corresponding random forest model.

The random forest method used in that study is broadly similar to the bagging method used in our study, in the sense that both build a model with a set of decision trees. However, each of the two random forests used by Lombardo et al. [13] had 500 trees, making the model more robust but also much slower to build and harder to be interpreted, by comparison with the smaller set of 10 trees in our Bagging model. In addition, the decision trees used in the random forest in Lombardo et al.’s work are regression trees, whilst in our work we used instead model trees, which had somewhat better predictive accuracy in our preliminary experiments. Concerning the threshold of 10 L/Kg used to define high and low Vss values for the MDA algorithm, this threshold choice seems very ad-hoc. However, to mitigate this problem and try to improve the prediction of Vss for compounds with Vss near the boundary of 10 L/Kg, the random forest predicting high Vss was built using training compounds with Vss ≥ 5 L/Kg, whilst the random forest predicting low Vss was built using as training set all available training compounds. Another difference with respect with our work is that we used only values of Vss in steady state for 604 compounds obtained from Obach et al.’s work [14], whilst in Lombardo et al.’s work [13] the dataset used was not only much smaller, with 384 compounds, but the Vss values for about 10% of compounds was the Vss during the terminal elimination phase, rather than in steady state. These dataset limitations are due to the fact that the study by Lombardo et al. predates the availability of the larger dataset of compounds with Vss in steady state made available by Obach et al..

The random forest method was also used by Berellini et al. [47], using a larger set of 669 compounds and using Vss in steady state available from Obach et al.’s work [14]. That study also built a random forest with 500 trees, with the aforementioned pro and cons.

Note that none of those two studies [13,47] reported a systematic interpretation of their random forest models (presumably due to the complexity of interpreting 500 trees); unlike this work, where the trees in a Bagging model were interpreted as discussed earlier.

### Outliers of Vss predictions

**Figure 2:**
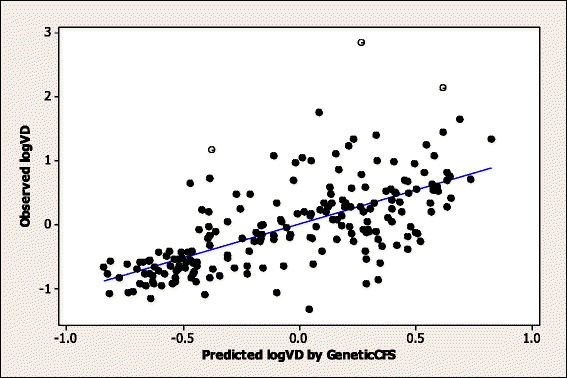
**Observed vs predicted log Vss for the external validation set using the model built by Bagging M5P from the descriptors selected by CFS; outliers have been identified by empty circles.**

In this study, the predicted tissue partitioning values and the molecular properties were able to provide a reasonable prediction of Vss for the majority of the drugs in the external validation set. However, there are a number of outlier compounds with large deviation between the measured and predicted Vss values. Figure 2 shows the plot of observed log Vss versus log Vss predicted by the best model built by Bagging M5P from the descriptors selected by CFS for the external validation set. Broadly speaking, most outliers are compounds with high Vss values that were substantially under-predicted by all the three best models analyzed earlier in the Discussion section. For example, out of 15 compounds with highest Vss values in the external dataset (11–700 L/Kg), 12 have been underpredicted with a GMFE > 4 by all the three models. This phenomenon of under-prediction of high Vss values was also observed, e.g., in Lombardo et al.’s work [13].

Two outlier compounds whose Vss were substantially under-predicted by our models are hydroxychloroquine and chloroquine, which are antimalarial drugs. These drugs have the very high Vss values of 700 L/Kg and 140 L/Kg, respectively, which are much higher than the highest Vss of compounds in the training set used to build our models. Hence, it is not surprising they are underpredicted by our models. Actually, these two drugs were removed from the dataset used by del Amo et al. [45], which avoided their negative influence in the measure of predictive accuracy of the models in that work, but we preferred not to remove any compound from the dataset based on its prediction difficulty. An explanation for the very high Vss of chloroquine is that it accumulates in lysosomes due to an ion-trapping mechanism [48,49]. Another outlier whose Vss was substantially under-predicted in our models is artesunate. Although the Vss of artesunate is 15 L/Kg, its median Vss value obtained for a group of 11 patients with malaria varied from 2.2 to 39 L/Kg [22].

In general, possible explanations for other underpredicted outliers in our models could be that they undergo active transport (by influx or efflux transporters) or exhibit specific binding to some tissues, or they are stored in subcellular compartments in specific tissues. For example, ion-trapping is a mechanism driven by pH gradients that leads to weakly basic drugs accumulation in lysosomes or other acidic membrane-bound intracellular compartments [50]. In addition, some lipophilic weakly basic drugs induce “phospholipidosis”, a phenomenon characterized by histological changes in certain body tissues as a result of the formation of many phospholipid- and cholesterol-rich multivesicular bodies and multilamellar bodies that accumulate large quantities of the drug and phospholipids [48,51]. However, as pointed out by del Amo et al. [45], it is difficult to know if active transport and specific binding occur to an extent that is large enough to significantly hinder the prediction of Vss, and a precise explanation of outliers would require extensive experimental work, which is out of the scope of this paper.

## Conclusions

In this work we have used mainly decision tree-based regression methods, but also a feature selection method (correlation-based feature selection with genetic search), to build regression models that predict the Vss of chemical compounds based on those compounds’ molecular descriptors and predicted tissue:plasma partition coefficients (K_t:p_) in rat.

In order to predict Vss, we investigated three approaches: (a) predicting Vss from both the predicted K_t:p_ values and molecular descriptors; (b) predicting Vss from the molecular descriptors only; (c) a two-step approach, where we first used the correlation-based feature selection (CFS) method to select a subset of relevant features from both predicted K_t:p_ values and molecular descriptors, and next we predicted Vss from the set of features selected by the CFS method. In our experiments, the results indicated that the use of predicted K_t:p_ values as descriptors could be beneficial for predicting Vss if prior feature selection is applied (approach (c)). In addition, our results indicate that different decision tree methods and workflows can be used for Vss prediction and we have shown an example of a model developed using K_t:p_ for adipose tissue.

We also compared our work with several other works predicting Vss in the literature, concluding that, although some of those works obtained smaller prediction errors, in general they focused on a smaller set of compounds with a narrower applicability domain, by comparison with the larger, more diverse set of compounds used in this work. The mean fold error of our selected model (approach (c)) was 2.29. This is a reasonable accuracy when considering the mean fold error reported in the literature for the interspecies scaling of 1.56 – 2.78 [52] and an animal to human extrapolation of Vss for a small range of drugs showing an average error of 1.82 [21]. The results of this investigation identified a problem with the prediction of Vss for a number of compounds (outliers). An analysis of the outliers indicates that these are mainly compounds with extremely large volumes of distribution. Such extreme volumes of distribution are generally the results of compounds accumulating in specific organs due to specific interactions with certain tissue constituents, transporter proteins or storage in subcellular compartments. Availability of tissue partition coefficient data for a larger number of compounds in the future should lead to more accurate predictions of K_t:p_s and consequently a better prediction of Vss from such data. In addition, other mechanisms leading to extreme Vss values and the subcellular transport properties of drugs can be investigated in order to aid the accurate prediction of Vss [48].

## Experimental

All data mining computations were done with the free data mining software WEKA 3.6 [24]. The methods included variations of decision trees like model trees, REP-Tree, regression trees and M5-Rules built by the M5P algorithm and the Bagging-M5P method with the default value of 10 for the number of trees.

## Additional file

Additional file 1:
**The following additional data are available with the online version of this paper.** Additional file 1 is a table in MS Excel format listing experimentally derived K_t:p_ values collated from the literature for a number of rat tissues for 110 compounds including the original references for the data.

## References

[1] Rosenbaum SE (2011). Basic pharmacokinetics and pharmacodynamics.

[2] De Buck SS, Sinha VK, Fenu LA, Gilissen RA, Mackie CE, Nijsen MJ (2007). The prediction of drug metabolism tissue distribution and bioavailability of 50 structurally diverse compounds in rat using mechanism-based absorption distribution and metabolism prediction tools. Drug Metab Dispos.

[3] Ghafourian T, Barzegar-Jalali M, Dastmalchi S, Khavari-Khorasami T, Hakimiha N, Nokhodchi A (2006). QSPR models for the prediction of apparent volume of distribution. Int J Pharm.

[4] Di L, Feng B, Goosen TC, Lai Y, Steyn SJ, Varma MV (2013). A perspective on the prediction of drug pharmacokinetics and disposition in drug research and development. Drug Metab Dispos.

[5] Louis B, Agrawal VK (2012). Quantitative Structure-Pharmacokinetic Relationship (QSPkR) analysis of the volume of distribution values of anti-infective agents from J group of the ATC classification in humans. Acta Pharm.

[6] Berry LM, Roberts J, Be X, Zhao Z, Lin M-HJ (2010). Prediction of V_ss_ from *in vitro* tissue-binding studies. Drug Metab Dispos.

[7] Duffy JC, Cronin MTD, Livingstone DJ (2004). Prediction of pharmacokinetic parameters in drug design and toxicology. Predicting chemical toxicity and fate.

[8] Graham H, Walker M, Jones O, Yates J, Galetin A, Aarons L (2012). Comparison of in-vivo and in-silico methods used for prediction of tissue:plasma partition coefficients in rat. J Pharm Pharmacol.

[9] Maguire TJ, Novik E, Chao P, Barminko J, Nahmias Y, Yarmush ML (2009). Design and application of microfluidic systems for *in vitro* pharmacokinetic evaluation of drug candidates. Curr Drug Metab.

[10] Jones RD, Jones HM, Rowland M, Gibson CR, Yates JWT, Chien JY (2011). PhRMA CPCDC initiative on predictive models of human pharmacokinetics part 2: comparative assessment of prediction methods of volume of distribution. J Pharm Sci.

[11] Madan AK, Dureja H, Reisfeld B, Mayeno AN (2012). Prediction of pharmacokinetic parameters. Computational toxicology: volume I methods in molecular biology Vol 929.

[12] Xu C, Mager DE (2011). Quantitative structure–pharmacokinetic relationships. Expert Opin Drug Metab Toxicol.

[13] Lombardo F, Obach RS, DiCapua FM, Bakken GA, Lu J, Potter DM (2006). A hybrid mixture discriminant analysis – random forest computational model for the prediction of volume of distribution of drugs in human. J Med Chem.

[14] Obach RS, Lombardo F, Waters NJ (2008). Trend analysis of a database of intravenous pharmacokinetic parameters in humans for 670 drug compounds. Drug Metab Dispos.

[15] Poulin P, Ekins S, Theil FP (2011). A hybrid approach to advancing quantitative prediction of tissue distribution of basic drugs in human. Toxicol Appl Pharmacol.

[16] Bois FY, Jamei M, Clewell HJ (2010). PBPK modeling of inter-individual variability in the pharmacokinetics of environment chemicals. Toxicology.

[17] Poulin P, Theil F-P (2012). Prediction of pharmacokinetics prior to *in vivo* studies 1 mechanism based prediction of volume of distribution. J Pharm Sci.

[18] Peyret T, Poulin P, Krishnan K (2010). A unified algorithm for predicting partition coefficients for PBPK modeling of drugs and environment chemicals. Toxicol Appl Pharmacol.

[19] Poulin P, Haddad S (2012). Advancing prediction of tissue distribution and volume of distribution of highly lipophilic compounds from a simplified tissue-composition-based model as a mechanistic animal alternative method. J Pharm Sci.

[20] Lin JH, Sugiyama Y, Awazu S, Hanano M (1982). *In vitro* and *in vivo* evaluation of the tissue-to-blood partition coefficient for physiological pharmacokinetic models. J Pharmacokinetic Biopharm.

[21] Mahmood I (1998). Interspecies scaling: predicting volumes, mean residence time and elimination half-life some suggestions. J Pharm Pharmacol.

[22] Zhivkova Z, Doytchinova I (2012). Prediction of steady-state volume of distribution of acidic drugs by quantitative structure-pharmacokinetics relationships. J Pharm Sci.

[23] Limbu K (2013). Computational models for the estimation of volume of distribution. MPharm Thesis.

[24] Witten H, Frank E (2005). Data mining: practical machine learning tools and techniques.

[25] Freitas AA, Wieser DC, Apweiler R (2010). On the importance of comprehensible classification models for protein function prediction. IEEE/ACM Trans Comput Biol Bioinformatics.

[26] Freitas AA (2013). Comprehensible classification models – a position paper. ACM SIGKDD Explorations.

[27] Quinlan JR (1993). C45: program for machine learning.

[28] Breiman L, Friedman JH, Olshen RA, Stone CJ (1984). Classification and regression tree.

[29] Quinlan JR (1992). Learning with continuous classes. Proceedings of the 5th Australian joint conference on artificial intelligence.

[30] Holmes G, Hall M, Frank E (1999). Generating rule sets from model trees. Proceedings of the twelfth Australian joint conference on artificial intelligence.

[31] Breiman L (1996). Bagging predictors. Mach Learn.

[32] Newby D, Freitas AA, Ghafourian T (2013). Pre-processing feature selection for improved C&RT models for oral absorption. J Chem Inform Model.

[33] Hall M (2000). A correlation-based feature selection for discrete and numeric class machine learning. Proceedings of the 17th international conference on machine learning (ICML-2000).

[34] Freitas AA (2002). Data mining and knowledge discovery with evolutionary algorithms.

[35] Demir-Kavuk O, Bentzien J, Muegge I, Knapp E-W (2011). DemQSAR: predicting volume of distribution and clearance of drugs. J Comput Aided Mol Des.

[36] Kato Y, Hirate J, Sakaguchi K, Ueno M, Horikoshi I (1987). Age-dependent changes in phenytoin tissue bindings in rats: comparison between *in vivo* and *in vitro* tissue-to-blood partition coefficients (Kp values) of phenytoin. J Pharmacobiodyn.

[37] Clausen J, Bickel MH (1993). Prediction of drug distribution in distribution dialysis and *in vivo* from binding to tissues and blood. J Pharm Sci.

[38] Katritzky AR, Petrukhin R, Tatham D, Basak S, Benfenati E, Karelson M (2001). Interpretation of quantitative structure property and -activity relationships. J Chem Inf Comput Sci.

[39] Poulin P, Theil F-P (2009). Development of a novel method for predicting human volume of distribution at steady-state of basic drugs and comparative assessment with existing methods. J Pharm Sci.

[40] Wildman SA, Crippen GM (1999). Prediction of physiochemical parameters by atomic contributions. J Chem Inf Comput Sci.

[41] Ghafourian T, Barzegar-Jalali M, Hakimiha N, Cronin MT (2004). Quantitative structure-pharmacokinetic relationship modelling: apparent volume of distribution. J Pharm Pharmacol.

[42] Clark DE (1999). Rapid calculation of polar molecular surface area and its application to the prediction of transport phenomena 1 prediction of intestinal absorption. J Pharm Sci.

[43] Ghafourian T, Freitas AA, Newby D (2012). The impact of training set data distributions for modelling of passive intestinal absorption. Int J Pharm.

[44] Gasteiger J, Marsili M (1980). Iterative partial equalization of orbital electronegativity - a rapid access to atomic charges. Tetrahedron.

[45] del Amo EM, Ghemtio L, Xhaard H, Yliperttula M, Urtti A, Kidron H (2013). Applying linear and non-linear methods for parallel prediction of volume of distribution and fraction of unbound drug. PLoS One.

[46] Paixão P, Aniceto N, Gouveia LF, Morais JA (2014). Prediction of drug distribution in rat and humans using an artificial neural networks ensemble and a PBPK model. Pharm Res.

[47] Berellini G, Springer C, Waters NJ, Lombardo F (2009). In silico prediction of volume of distribution in human using linear and nonlinear models on a 669 compound data set. J Med Chem.

[48] Zheng N, Zhang X, Rosania GR (2011). Effect of Phospholipidosis on the Cellular Pharmacokinetics of Chloroquine. J Pharmacol Exp Therapeut.

[49] Min KA, Zhang X, Yu JY, Rosania GR (2014). Computational approaches to analyse and predict small molecule transport and distribution at cellular and subcellular levels. Biopharm Drug Dispos.

[50] Gong Y, Zhao Z, McConn DJ, Beaudet B, Tallman M, Speake JD (2007). Lysosomes contribute to anomalous pharmacokinetic behavior of melanocortin-4 receptor agonists. Pharm Res.

[51] Yudate HT, Kai T, Aoki M, Minowa Y, Yamada T, Kimura T (2012). Identification of a novel set of biomarkers for evaluating phospholipidosis-inducing potential of compounds using rat liver microarray data measured 24-h after single dose administration. Toxicology.

[52] Obach RS, Baxter JG, Liston TE, Silber BM, Jones BC, MacIntyre F (1997). The prediction of human pharmacokinetic parameters from preclinical and *in vitro* metabolism data. J Pharmacol Exp Ther.

